# Plant-Derived *Trans*-β-Caryophyllene Boosts Glucose Metabolism and ATP Synthesis in Skeletal Muscle Cells through Cannabinoid Type 2 Receptor Stimulation

**DOI:** 10.3390/nu13030916

**Published:** 2021-03-12

**Authors:** Federica Geddo, Susanna Antoniotti, Giulia Querio, Iris Chiara Salaroglio, Costanzo Costamagna, Chiara Riganti, Maria Pia Gallo

**Affiliations:** 1 Department of Life Sciences and Systems Biology, University of Turin, Via Accademia Albertina 13, 10123 Turin, Italy; federica.geddo@unito.it (F.G.); susanna.antoniotti@unito.it (S.A.); giulia.querio@unito.it (G.Q.); 2 Department of Oncology, University of Turin, Via Santena 5/bis, 10126 Turin, Italy; irischiara.salaroglio@unito.it (I.C.S.); costanzo.costamagna@unito.it (C.C.); chiara.riganti@unito.it (C.R.)

**Keywords:** *trans*-β-caryophyllene, cannabinoid type 2 receptor, glucose metabolism, glucose uptake

## Abstract

Skeletal muscle plays a pivotal role in whole-body glucose metabolism, accounting for the highest percentage of glucose uptake and utilization in healthy subjects. Impairment of these key functions occurs in several conditions including sedentary lifestyle and aging, driving toward hyperglycemia and metabolic chronic diseases. Therefore, strategies pointed to improve metabolic health by targeting skeletal muscle biochemical pathways are extremely attractive. Among them, we focused on the natural sesquiterpene and cannabinoid type 2 (CB2) receptor agonist *Trans-*β-caryophyllene (BCP) by analyzing its role in enhancing glucose metabolism in skeletal muscle cells. Experiments were performed on C2C12 myotubes. CB2 receptor membrane localization in myotubes was assessed by immunofluorescence. Within glucose metabolism, we evaluated glucose uptake (by the fluorescent glucose analog 2-NBDG), key enzymes of both glycolytic and oxidative pathways (by spectrophotometric assays and metabolic radiolabeling) and ATP production (by chemiluminescence-based assays). In all experiments, CB2 receptor involvement was tested with the CB2 antagonists AM630 and SR144528. Our results show that in myotubes, BCP significantly enhances glucose uptake, glycolytic and oxidative pathways, and ATP synthesis through a CB2-dependent mechanism. Giving these outcomes, CB2 receptor stimulation by BCP could represent an appealing tool to improve skeletal muscle glucose metabolism, both in physiological and pathological conditions.

## 1. Introduction

Aside from the many important biological functions such as insulating the internal organs, maintaining core body temperature, and supporting movement, skeletal muscle is a key site for glucose uptake and storage and plays a critical role in maintaining systemic glucose homeostasis through an interactive cross-talk with hepatic and adipose tissue [1,2]. Insulin is the major stimulator of glucose uptake in skeletal muscle mediated by the insulin-sensitive glucose transporter protein 4 (GLUT4) translocation from an intracellular location to the plasma membrane [3]. Since glucose transport into skeletal muscle regulates blood glucose concentration, it is a critical step in insulin-regulated glucose metabolic pathways such as glycolysis and glycogen synthesis. Dysfunctions in this process represent an important defect in the insulin action [2,4]. Impairment of glucose metabolism can occur in a range of conditions, from skeletal muscle disease like sarcopenia and cachexia, which are associated with a reduction in the amount of available muscle mass enough to maintain insulin-stimulated glucose uptake, to sedentary lifestyle and chronic metabolic diseases such as diabetes and obesity, characterized by the onset of insulin resistance [1,5,6,7]. This impairment is particularly dangerous in cachectic cancer patients, where the low skeletal muscle mass could induce insulin resistance and decrease glucose clearance from the blood, resulting in more glucose disposable for the uptake by tumor cells, predisposing to poor clinical outcomes [8].

In these contexts, interventions aimed at improving skeletal muscle metabolism by counteracting the insulin resistance and preserving a healthy muscle mass represent appealing wholesome approaches. Among the therapeutic strategies fitting this goal, nutritional supplementation could account for an efficient weapon mimicking insulin and/or physical exercise effects.

In the landscape of plant-derived compounds assessed as dietary supplements, we draw our attention to the natural bicyclic sesquiterpene *trans*-β-caryophyllene (BCP), a plant volatile compound found in large amounts in the essential oil of different spices and food plants such as hemp (*Cannabis sativa* L.), oregano (*Origanum vulgare* L.), cinnamon (*Cinnamomum* spp.), black pepper (*Piper nigrum* L.), and cloves (*Syzygium aromaticum* L.) is approved by the Food and Drug Administration and the European Food Safety Authority as a flavoring agent, and commercially used as a food additive and in the cosmetic industry/production [9,10]. Pharmacologically, BCP acts as a full selective functional agonist of the cannabinoid type 2 (CB2) receptor, thus behaving as a phytocannabinoid. Notably, this specificity (Ki = 32.1 nM) of BCP to the CB2 receptor, which is primarily expressed in peripheral tissues, implies that its action lacks the typical psychoactive effects associated with the activation of the cannabinoid type 1 (CB1) receptor, mainly expressed in the central nervous system, supporting its potential use in medicine [9,11]. Indeed, many studies have reported the beneficial anti-cancer [12,13], neuroprotective [14,15], cardioprotective [16,17], anti-oxidant, and anti-inflammatory [11] activities of BCP. Furthermore, BCP has a great impact on metabolic diseases such as obesity [18,19], hepatic steatosis [19,20], and type 2 diabetes mellitus [19,21,22].

We have previously demonstrated that a plant extract rich in BCP increases glucose uptake and promotes GLUT4 translocation from the cytoplasm to the membrane in C2C12 myotubes [23]. Therefore, in the present study, we deepen the role of BCP in enhancing glucose metabolism by attesting its involvement in the boost of glycolytic and oxidative pathways and ATP production. Moreover, we investigated the CB2 receptor involvement in mediating both glucose uptake and glucose metabolism by testing CB2 receptor localization on plasma membranes of C2C12 myotubes and by employing the CB2 antagonists AM630 and SR144528 in both glucose uptake and biochemical measurements.

## 2. Materials and Methods

### 2.1. Reagents

*Trans*-β-Caryophyllene (BCP) was kindly provided by Biosfered srl (Turin, Italy) as Endophyllene^®^-P FL (batch PNF01-2003001), a black pepper (*Piper nigrum* L.) liquid extract that contains 80% *w*/*w* of BCP in rice oil, corresponding to a 3.5 M concentration of BCP; for experiments, a stock solution of 1 M in Dimethyl Sulfoxide Solvent (DMSO) was obtained, then diluted in culture medium for cell treatments. Concentrations reported in this work refer to those of BCP in each dilution.

2-[*N*-(7-Nitrobenz-2-oxa-1,3-diazol-4-yl)amino]-2-deoxy-d-glucose (2-NBDG) was obtained from Invitrogen (Carlsbad, CA, USA); anti CB2R primary antibody and CB2R blocking peptide were from Cayman Chemical (Ann Arbor, MI, USA); and anti-rabbit secondary antibody Alexa Fluor 568 was from Thermo Fisher Scientific (Waltham, MA, USA). CB2R antagonists AM630 and SR144528 were obtained from Cayman Chemicals and dissolved in DMSO at 5 and 10 mM stock solution, respectively.

Unless otherwise specified, all chemicals were purchased from Sigma Aldrich (St. Louis, MO, USA).

### 2.2. Cell Cultures

The mouse myoblast cell line C2C12 (ECACC 91031101, lot 17I044) was purchased from the European Collection of Authenticated Cell Cultures (ECACC, Salisbury, UK) and cultured in high-glucose Dulbecco’s Modified Eagle Medium (DMEM) supplemented with 10% Fetal Bovine Serum (FBS), 1% penicillin/streptomycin, and 2 mM L-glutamine in a humidified atmosphere of 5% CO_2_ at 37 °C. Cultures were plated at a density of 2 × 10^3^ cells per cm^2^ on tissue plastic dishes (Becton Dickinson, Franklin Lakes, NJ, USA) and sub-cultured before reaching 70% confluence. For confocal microscopy experiments, cells were seeded at a density of 10 × 10^3^ cell/cm^2^ on coverslips or glass bottom dishes (VWR Int., Radnor, PA, USA) to enhance adhesion. After cells reached confluence, differentiation was induced by changing the medium to DMEM supplemented with 2% horse serum (HS) for an additional five to seven days. The day before the experiments, C2C12 cells were starved for 24 h in glucose and serum free DMEM.

### 2.3. CB2 Receptor (CB2R) Immunofluorescence Staining

C2C12 cells were grown and differentiated on glass coverslips. After 24 h without glucose and serum, cells were fixed for 40 min in 4% paraformaldehyde dissolved in 0.01 M phosphate buffer, pH 7.4. After three washes with Phosphate Buffered Saline (PBS), cells were incubated for 20 min with 0.3% *v*/*v* Triton X100 and 1% *w*/*v* bovine serum albumin (BSA) in PBS and stained for 24 h at 4 °C with the primary rabbit polyclonal antibody anti-CB2R, 1:100. Cover slides were washed twice with PBS and incubated 1 h at room temperature with the secondary antibody, anti-rabbit Alexa 200 Fluor 568, 1:1000. After two washes in PBS, coverslips were mounted on standard slides with Prolong Gold Antifade mountant (Thermo Fisher) and observed after 48 h under confocal microscope. Specificity of CB2R staining was confirmed by incubating the primary antibody with its blocking peptide (1/1 ratio), directed against the sequence between N-terminus and the first transmembrane domain of the human CB2 protein for 1 h at room temperature. Aspecific staining with secondary antibody alone was checked. Fluorescence images at 568 nm were acquired using an Olympus Fluoview 200 laser scanning confocal system (Olympus America Inc., Melville, NY, USA) mounted on an inverted IX70 Olympus microscope, equipped with a 60X Uplan FI (NA 1.25) oil-immersion objective. Fluorescence qualitative variations were evaluated with the definition and measurement of regions of interest (ROIs) using the ImageJ software (Rasband, W.S., U.S. National Institutes of Health, Bethesda, MA, USA, 1997–2018, https://imagej.nih.gov/ij/, accessed on 15 February 2021).

### 2.4. Glucose Uptake Measurements

C2C12 cells differentiated on glass bottom dishes, after 24 h without glucose and serum, were treated with BCP 10 nM, BCP together with CB2R inhibitors AM630 or SR144528, both at 5 μM (after a 2 h pretreatment) or CB2R inhibitors alone (2 h pretreatment) and simultaneously loaded with 100 μM of 2-NBDG in glucose and serum free DMEM, for 30 min in the dark. Insulin (25 nM) was used as a positive control. After two washes in PBS, cells were observed in confocal microscopy. Fluorescence images at 488 nm were acquired using an Olympus Fluoview 200 laser scanning confocal system (Olympus America Inc., Melville, NY, USA) mounted on an inverted IX70 Olympus microscope, equipped with a 60X Uplan FI (NA 1.25) oil-immersion objective.

Fluorescence variations were calculated with the definition and measurement of regions of interest (ROIs) using the ImageJ software (Rasband, W.S., U.S. National Institutes of Health, Bethesda, MA, USA, 1997–2018, https://imagej.nih.gov/ij/, accessed on 15 February 2021). Data from six independent experiments for AM630 and four independent experiments for SR144528 were evaluated as mean fluorescence and expressed as percentage referred to control condition; these values were then summarized to calculate mean ± standard error of the mean (SEM).

### 2.5. Glycolysis Enzyme Activity

C2C12 myotubes, grown and differentiated on plastic Petri dishes, were starved for 24 h then treated for 30 min with BCP alone or in combination with CB2R antagonists like for the glucose uptake experiments. Cells were washed with fresh PBS, detached with trypsin/EDTA, re-suspended at 1 × 10^5^ cells/mL in 0.2 mL of 100 mM TRIS/1 mM EDTA (pH 7.4), and sonicated on ice with two 10 s bursts. Enzymatic activities were measured on 10 µL cell lysates, incubated for 5 min at 37 °C. The protein content was measured using the BCA1 Kit (Sigma Aldrich). The activity of phosphofructokinase-1 (PFK1) was measured spectrophotometrically as reported in Sharma (2011) [24]. The activity of glyceraldehyde 3-phosphate dehydrogenase (GAPDH) and enolase were measured spectrophotometrically according to Beutler (1975) [25]. Pyruvate kinase (PK) activity was measured by using the Hexokinase Colorimetric Assay Kit (Sigma Aldrich). For all enzymes, the activity was monitored measuring the absorbance variation at 340 nm using a Synergy HTX 96-well microplate reader (Bio-Tek Instruments, Winooski, VT). The kinetics were linear throughout the measurement. Results were expressed as nmoles NAD^+^/min/mg cell proteins (PFK-1, enolase, PK) or nmoles NADH/min/mg cell proteins (GAPDH).

### 2.6. Mitochondria Extraction

Mitochondrial fractions were isolated as previously reported [26], with minor modifications. Samples were lysed in 0.5 mL buffer A (50 mM Tris, 100 mM KCl, 5 mM MgCl_2_, 1.8 mM ATP, 1 mM EDTA, pH 7.2), supplemented with protease inhibitor cocktail III (Sigma Chemical Co), 1 mM phenylmethylsulfonyl fluoride, and 250 mM NaF. Samples were clarified by centrifuging at 650× *g* for 2 min at 4 °C, and the supernatant was collected and centrifuged at 13,000× *g* for 5 min at 4 °C. This supernatant was discarded and the pellet containing mitochondria was washed in 0.5 mL buffer A and resuspended in 0.25 mL buffer B (250 mM sucrose, 15 mM K_2_HPO_4_, 2 mM MgCl_2_, 0.5 mM EDTA, 5% *w*/*v* BSA). A 50 μL aliquot was sonicated and used for the measurement of protein content or western blotting; the remaining part was stored at −80 °C until use. To confirm the presence of mitochondrial proteins in the extracts, 10 μg of each sonicated sample were subjected to SDS-PAGE and probed with an anti-porin antibody (Abcam, Cambridge, UK; data not shown). To exclude any mitochondrial contamination in the cytosolic extracts, the absence of porin in the latter was analyzed by immunoblotting (data not shown).

### 2.7. Pyruvate Dehydrogenase (PDH) and Tricarboxylic Acid (TCA) Enzymes Activity

The enzymatic activities of PDH, citrate synthase, aconitase, isocitrate dehydrogenase, α-ketoglutarate (αKG) dehydrogenase, and succinate dehydrogenase were measured on 10 µg mitochondrial proteins using the Pyruvate Dehydrogenase (PDH) Assay Kit (Abcam), the Citrate Synthase Assay Kit (Sigma Aldrich), the Aconitase Assay Kit (Cayman Chemical), the Isocitrate Dehydrogenase Assay Kit (Sigma Aldrich), the Alpha Ketoglutarate (alpha KG) Assay Kit (Abcam), and the Succinate Dehydrogenase Activity Colorimetric Assay Kit (BioVision, Milpitas, CA), as per the manufacturer’s instructions. Results were expressed as nmoles NADH/mg mitochondrial proteins (PDH, isocitrate dehydrogenase, αKG dehydrogenase), nmoles citrate or isocitrate/mg mitochondrial proteins (citrate synthase, aconitase), and nmoles FADH_2_/mg mitochondrial proteins (succinate dehydrogenase).

### 2.8. Electron Transport Chain (ETC) and Mitochondrial ATP

To measure the electron flux from complex I to complex III, taken as the index of the mitochondrial respiratory activity, 50 μg of non-sonicated mitochondrial samples were resuspended in 0.2 mL buffer A (5 mmol/L KH_2_PO_4_, 5 mmol/L MgCl_2_, 5% *w*/*v* bovine serum albumin) and 0.1 mL buffer B (25% *w*/*v* saponin, 50 mmol/L KH_2_PO_4_, 5 mmol/L MgCl_2_, 5% *w*/*v* bovine serum albumin, 0.12 mmol/L cytochrome c-oxidized form, 0.2 mmol/L NaN_3_) was added for 5 min at room temperature. The reaction was started with 0.15 mmol/L NADH and was followed for 5 min, reading the absorbance at 550 nm by a Packard microplate reader EL340 (Bio-Tek Instruments). The results were expressed as nanomoles of cytochrome c reduced/min/mg mitochondrial proteins [26]. Mitochondrial ATP levels were measured using the ATP Bioluminescent Assay Kit (Sigma-Aldrich). ATP was quantified as relative light units (RLU) and converted into nmoles ATP/mg mitochondrial proteins, according to the calibration curve previously set.

### 2.9. Statistical Analysis

Data were expressed as mean ± standard error of the mean (SEM); statistical analysis was performed using ANOVA (one-way analysis of variance) followed by Bonferroni’s multiple comparison test. Differences with *p* < 0.05 were considered statistically significant.

## 3. Results

### 3.1. CB2 Receptor Is Expressed in C2C12 Skeletal Muscle Cells

To confirm the CB2 receptor localization in C2C12 myotubes, we performed immunofluorescence experiments. Differentiated cells were incubated with the CB2 receptor antibody or CB2 receptor antibody previously treated with its blocking peptide.

**Figure 1:**
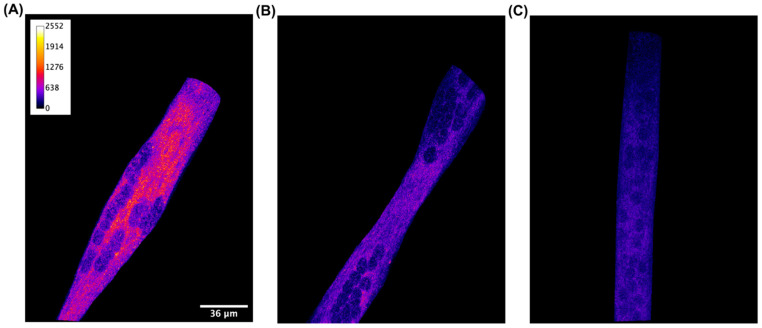
Qualitative detection with immunofluorescence staining of CB2 receptor in C2C12 skeletal muscle cells. Confocal image of a representative XY acquisition (60× magnification) of (**A**) differentiated cells incubated with primary CB2 receptor antibody, (**B**) differentiated cells incubated with CB2 receptor antibody and its blocking peptide (1/1 ratio), and (**C**) differentiated cells incubated with secondary antibody alone. Scale bar: 36 μm. Secondary antibody, anti-rabbit Alexa 200 Fluor 568, 1:1000. Images are presented in pseudocolor (LUT = fire) to better show the fluorescent intensity variations in the range 0–2552.

As shown in Figure 1A, the CB2 receptor is detectable when C2C12 cells are incubated with the CB2 receptor antibody, while the fluorescent signal becomes significantly reduced when the antibody is supplied simultaneously with the blocking peptide (Figure 1B). This result confirms the presence of the CB2 receptor on C2C12 muscle cells.

### 3.2. CB2 Receptor Mediates Glucose Uptake in C2C12 Myotubes

As we have previously demonstrated that BCP improves glucose uptake in C2C12 muscle cells [23], here, we investigated how this effect requires CB2 receptor stimulation; for this purpose, we employed both AM630 and SR144528 as CB2 receptor antagonists in the glucose-uptake measurements. The experimental conditions tested on the 2-NBDG loaded cells were as follows: BCP 10 nM, BCP 10 nM + AM630 5 µM, AM630 5 µM, BCP 10 nM + SR144528 5 µM, and SR155528 5 µM; all treatments were performed for 30 min in the dark. Insulin 25 nM was used as a positive control of the glucose uptake.

**Figure 2:**
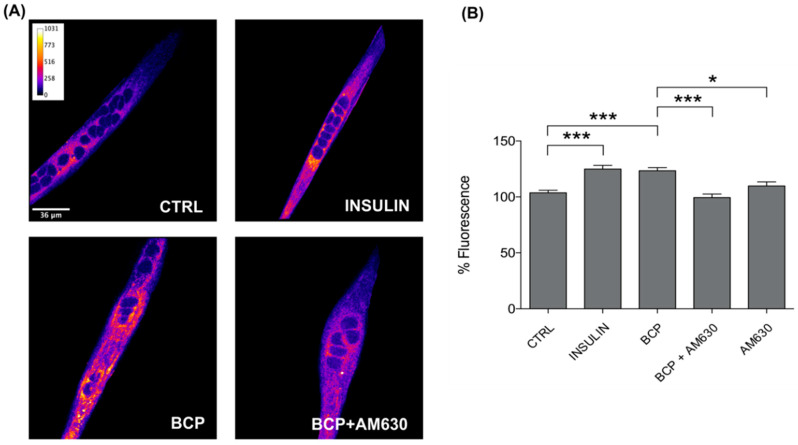
*Trans-*β-Caryophyllene (BCP) stimulates glucose uptake by CB2 receptor stimulation. (**A**) Representative confocal images of C2C12 incubated with the fluorescent glucose analog (2-NBDG) for 30 min in the dark. Images are presented in pseudocolor (LUT = fire) to better show the fluorescent intensity variations in the range 0–1031. Insulin was used as a positive control. Scale bar: 36 μm. (**B**) Bar graph summarizing glucose uptake experiments. CTRL: 103.66 ± 2.23, n cells = 129; Insulin: 124.98 ± 3.19, n cells = 133; BCP: 123.47 ± 2.76, n cells = 134; BCP + AM630: 99.43 ± 3.08, n cells = 86; AM630: 109.85 ± 3.54, n cells = 94. Data are represented as mean ± SEM (*n* = 6 independent experiments). * *p* < 0.05; *** *p* < 0.001.

**Figure 3:**
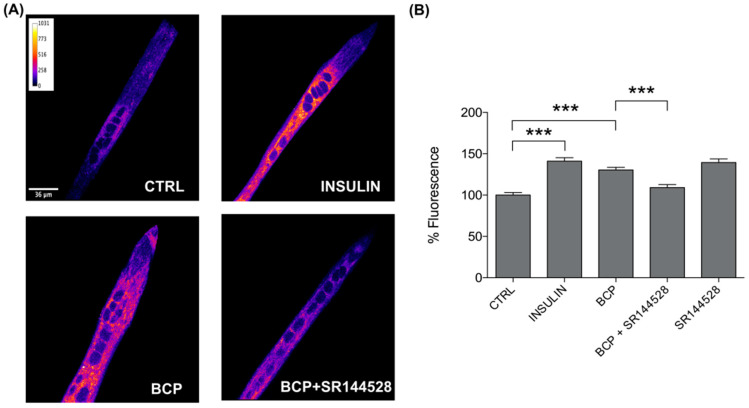
BCP enhances glucose uptake by CB2 receptor stimulation. (**A**) Representative confocal images of C2C12 incubated with the fluorescent glucose analog (2-NBDG) for 30 min in the dark. Images are presented in pseudocolor (LUT = fire) to better show the fluorescent intensity variations in the range 0–1031. Insulin was used as a positive control. Scale bar: 36 μm. (**B**) Bar graph summarizing glucose uptake experiments. CTRL: 100.00 ± 3.15, n cells = 66; Insulin: 141.06 ± 4.17, n cells = 60; BCP: 130.30 ± 3.16, n cells = 67; BCP + SR144528: 109.04 ± 3.64, n cells = 65; SR144528: 139.45 ± 4.26, n cells = 44. Data are represented as mean ± SEM (*n* = 4 independent experiments). *** *p* < 0.001.

As expected, a significant increase in glucose uptake was confirmed in C2C12 cells treated with BCP 10 nM, and, as a novelty, we observed a noticeable reduction in the uptake when BCP was co-administered with both AM630 5 µM (Figure 2) and SR144528 5 µM (Figure 3). We can therefore state the obligatory role of CB2 receptors in mediating the BCP-dependent stimulation of glucose uptake in skeletal myotubes. The increase in glucose uptake observed in the presence of SR144528 alone could be placed in the complex context of the CB receptor ligands, in which several CB receptor agonist and antagonists have been pointed out as inducers of CB1/CB2 receptor-independent effects [27].

### 3.3. Trans-β-Caryophyllene (BCP) Improves Glycolytic Metabolism

**Figure 4:**
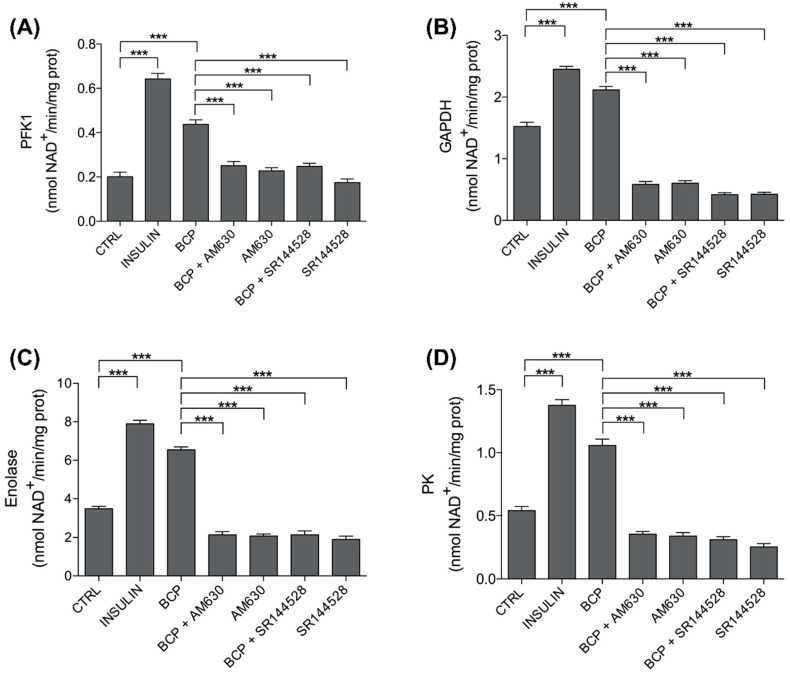
Effects of *trans*-β-carophyllene on glycolysis. C2C12 cells were left untreated (CTRL) or treated with insulin (25 nM for 30 min; INS), BCP (10 nM for 30 min), AM630 (5 µM for 2 h) or SR144528 (5 µM for 2 h), alone or in combination with 10 nM BCP, added in the last 30 min. The enzymatic activities of (**A**) phospho-fructokinase 1 (PFK1), (**B**) glyceraldehyde 3-phosphate dehydrogenase (GAPDH), (**C**) enolase, and (**D**) pyruvate kinase (PK) were measured spectrophotometrically in the whole cell lysates in triplicate. Data are expressed as means ± SEM (*n* = 3). *** *p* < 0.001.

To deepen the insulin-mimetic role of BCP in the myotube, we proceeded with our study toward the different steps of glucose metabolism. It is well proven that, after increasing the glucose uptake, insulin promotes glucose metabolism through glycolysis by increasing the activity of PFK2, GAPDH, enolase, and PK [28,29]; this was confirmed, as expected, in our experiments on C2C12 skeletal muscle cells stimulated for 30 min with Insulin 25 nM, a concentration in the physiological range (Figure 4). More importantly, BCP (10 nM for 30 min) acts as a strong insulin-mimetic, since it significantly increased the activity of all these glycolytic enzymes in C2C12 cells (Figure 4).

To understand if the BCP signaling pathway is also dependent on CB2 receptor in regulating the activity of enzymes involved in glycolysis, the latter was evaluated in C2C12 cells first pretreated with CB2 receptor antagonists, AM630 and SR144528 (5 µM, preincubation for 2 h), then stimulated with BCP 10 nM. Both inhibitors abrogated the effects of BCP, suggesting that its insulin-mimetic effect was dependent on the CB2 receptor (Figure 4).

Values represented in Figure 4 are reported in Table 1.

### 3.4. BCP Improves Mitochondrial Metabolism: PDH and TCA Enzyme Activity

**Figure 5:**
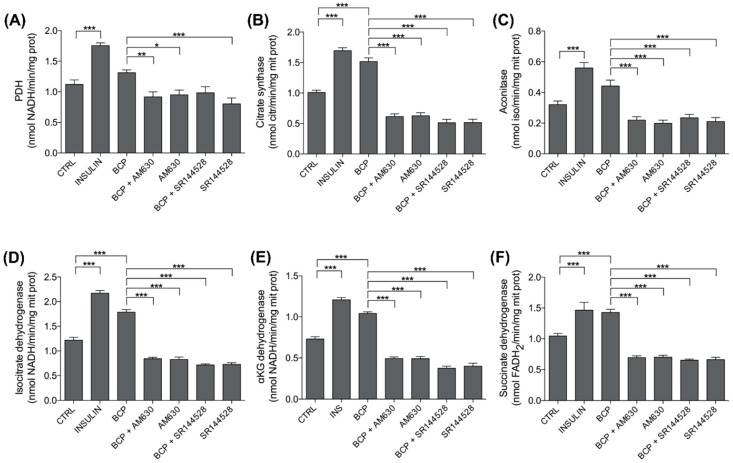
Effects of BCP on mitochondria metabolism. C2C12 cells were left untreated (CTRL) or treated with insulin (25 nM for 30 min; INS), BCP (10 nM for 30 min), AM630 (5 µM for 2 h), or SR144528 (5 µM for 2 h), alone or in combination with 10 nM BCP, added in the last 30 min. The enzymatic activities of (**A**) pyruvate dehydrogenase (PDH), (**B**) citrate synthase, (**C**) aconitase, (**D**) isocitrate dehydrogenase, (**E**) α-ketoglutarate (αKG) dehydrogenase, and (**F**) succinate dehydrogenase were measured spectrophotometrically in mitochondrial extracts in triplicate. Data are expressed as means ± SEM (*n* = 3). * *p* < 0.1, ** *p* < 0.01, *** *p* < 0.001.

Since muscle has a highly flexible metabolism oscillating between anaerobic and aerobic glycolysis, we next investigated the oxidative decarboxylation of pyruvate via PDH and its metabolism through the TCA cycle. As expected, insulin (25 nM, 30 min treatment) activated PDH and specific enzymes of the TCA cycle in C2C12 cells (Figure 5). Moreover, with the only exception of PDH and aconitase, where BCP produced a similar but less pronounced increase in the enzymatic activity than insulin, all the other TCA cycle enzymes increased by BCP at the same extent of insulin, thus confirming its insulin-mimetic effect (Figure 5). Co-administration of AM360 or SR144528 and BCP completely abrogated the effects of the latter, demonstrating the involvement of the CB2 receptor in the effects of BCP on glucose aerobic metabolism in mitochondria (Figure 5).

Values represented in Figure 5 are reported in Table 2.

### 3.5. BCP Improves Mitochondrial Metabolism: Electron Transport Chain (ETC) and Mitochondrial ATP

**Figure 6:**
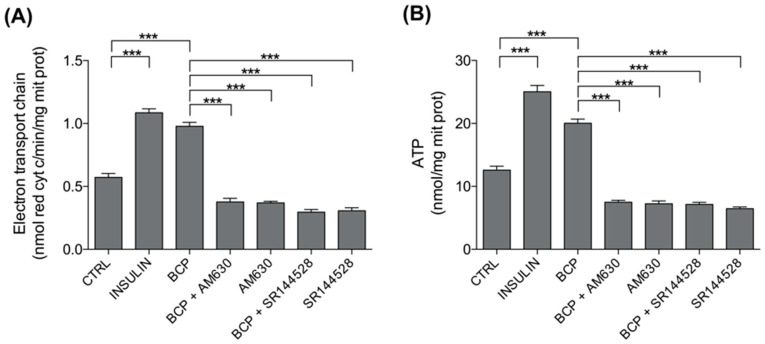
Effects of BCP on mitochondria metabolism. C2C12 cells were left untreated (CTRL) or treated with insulin (25 nM for 30 min; INS), BCP (10 nM for 30 min), AM630 (5 µM for 2 h) or SR144528 (5 µM for 2 h), alone or in combination with 10 nM BCP, added in the last 30 min. The electron flux from complex I to complex III (**A**) and the levels of ATP (**B**) were measured spectrophotometrically in mitochondrial extracts in triplicate. Data are expressed as means ± SEM (*n* = 3). *** *p* < 0.001.

The NADH produced in the TCA cycle is an excellent electron donor for the ETC and the consequent synthesis of ATP via oxidative phosphorylation (OXPHOS). In line with the effects on TCA cycle, both insulin and BCP strongly increased electron flux via ETC and the consequent ATP production, while CB2R antagonists AM360 or SR144528 both prevented the increase of ETC and ATP induced by BCP (Figure 6).

Values represented in Figure 6 are reported in Table 3.

## 4. Discussion

This study was designed to explore the beneficial role of the plant-derived sesquiterpene BCP on the skeletal muscle glucose metabolism from the viewpoint of a supplementary handling of the metabolic dysfunctions characterized by a failure in glycemic control and, more broadly, of the several pathophysiological contexts requiring optimal glucose fueling in skeletal muscles. In particular, we focused on the insulin-dependent glucose metabolic pathways, whose impairment is strictly associated with muscle mass loss and dysfunction [30].

Moreover, as in a previous report we demonstrated the efficacy of a BCP-enriched extract in improving glucose uptake in myotubes [23], the onward goal was to link this outcome, and further metabolic effects here analyzed, to the CB2 receptor pathway, expanding the relevance of the study toward cannabinoid system stimulation.

From this perspective, the first step in the project was the validation of the CB2 receptor expression in our cellular model. By immunofluorescence experiments, we clearly attested the CB2 receptor localization in C2C12 myotubes, laying the foundations for proceeding with the research. CB2 receptor expression in C2C12 myotubes was earlier highlighted by RT-PCR and western blot analysis [31]. Moreover, CB2 and CB1 receptor expression was also revealed in human and rodent skeletal muscle [32]. Functionally, while CB1 in skeletal muscle has been associated with a reduction in glucose uptake and fatty acid oxidation [33], Zheng et al. found that CB2 receptor stimulation in C2C12 myotubes by *trans*-β-caryophyllene promotes lipid oxidation through the sirtuin 1/peroxisome proliferator co-activator receptor 1α (SRT1/PGC1α) pathway [31], paving the way for a role of this receptor in lipid metabolism, and more generally in the management of obesity and insulin resistance. Besides these pathways specifically related to skeletal muscle, CB2 receptor stimulation has been recently underlined for its substantial contribution in the management of diabetes mellitus, thanks to its role in increasing insulin release and reducing inflammation and oxidative stress, thus ameliorating diabetic nephropathy, neuropathy, and retinopathy [34].

Fitting in this scenario, our next step was to test the CB2 receptor-dependence of the increase in glucose-uptake induced by BCP, which we previously showed [23]. As presented in both Figure 2 and Figure 3, BCP failed to improve the entry of glucose when C2C12 cells were treated with the CB2 receptor antagonists AM630 or SR144528. This is the first evidence of a CB2 dependent effect on glucose metabolism in a skeletal muscle model, thus adding a piece to the puzzle of the positive metabolic role of this receptor. Moreover, aside from a metabolic role, CB2 receptor activation in skeletal muscle has also been related to the inhibition of inflammation and fibrosis [35], and to a protective role against ischemia-reperfusion injury [36]. These previous studies were respectively carried out with the CB2 receptor inverse agonists Gp1a [35] and AM1241 [36], which are synthetic chemicals widely employed in both in vitro and in vivo models. In this context, BCP could represent a plant-derived option useful to stimulate CB2 receptors for both experimental purposes and nutraceutical applications.

The succeeding advancement of the study was planned to move forward with the metabolism of glucose by exploring the BCP-CB2-dependent modulation of glycolysis, aerobic glucose metabolism through mitochondrial intermediate metabolism, and ATP synthesis.

Firstly, we evaluated the effects on glycolysis of BCP, alone and in the presence of two CB2 receptor antagonists, AM360 and SR144528.

The results of this set of experiments are shown in Figure 4 and Table 1. As presented, the activity of PFK2, GAPDH, enolase, and PK were significantly increased by insulin, our positive control. This observation is in line with previous findings. Indeed, PFK2 [28] and PK [29] are known to be positively modulated by insulin. Moreover, in the heart, insulin stimulates the phosphorylation and activation of GAPDH [37], and the analysis of the glucose flux downstream 3-phosphoglycerate indicated that insulin upregulates enolase [38].

Similarly to insulin, BCP showed a stimulatory effect on glycolysis, confirming its role as a booster of glucose metabolism. Interestingly, and in line with previous observations carried out in pancreatic cancer cells [39], we also showed that the CB2 receptor inhibitors dampened the BCP-dependent stimulation of GAPDH activities and downstream enzymes enolase and PK.

As recent studies have underlined the role of glycolysis in promoting mTORC1 signaling and protein synthesis [40,41], thus improving skeletal muscle mass, this result is particularly appealing with respect to a potential benefit of BCP in the spectrum of the muscle-loss related dysfunctions.

In the second set of biochemical analysis, we evaluated the mitochondrial intermediate metabolism, in particular, the activity of PDH and specific enzymes of the TCA cycle (Figure 5 and Table 2).

Insulin stimulates PDH activity by repressing the inhibitory PDH kinase 4 [42]; moreover, PDH is critical in preserving skeletal muscle metabolic flexibility. In line with these observations, in C2C12 myotubes, insulin activated PDH and specific enzymes of the TCA cycle, which are controlled by insulin as suggested by their reduction in insulin-resistance, type 2 diabetes, and streptozotocin-induced diabetes: citrate synthase [43], aconitase [44], IDH [45], αKG dehydrogenase [46], and succinate dehydrogenase [47].

As observed for the glycolytic pathway, BCP displayed a stimulatory role also for PDH and the TCA cycle enzyme activity. Impaired PDH activity and reduced TCA cycle flux in skeletal muscle have been associated with obesity and type II diabetes as skeletal muscle is the primary site of systemic release of non-oxidized glycolytic products (lactate, pyruvate, alanine), which are biomarkers of chronic metabolic disease [48,49]. This evidence thus highlights the consequence of BCP stimulation on skeletal muscle homeostatic control of energy balance.

In Figure 5 and in Table 2, we also show that CB2 receptors are likely to be involved in the effect of BCP on TCA cycle stimulation. This result is the first evidence of a role of the cannabinoid system in the TCA cycle modulation, as previous data on pancreatic cancer cells showed limited inhibitory effects on the TCA cycle enzyme [39].

Finally, as a third biochemical evaluation, we focused on ETC, which is fueled by the NADH and FADH_2_ generated by the TCA cycle, and the consequent synthesis of ATP related to OXPHOS (Figure 6 and Table 3). Additionally, in this set of experiments, we demonstrated a stimulatory role of BCP that was CB2 receptor-dependent.

Limitation of this study could be addressed in the use of a murine model of skeletal muscle cells, but results obtained are promising for a possible translatability to human models.

A reduction in the synthesis of ATP in skeletal muscle leads to a decline in protein turnover, characterizing both aging-related functional changes and insulin-resistance conditions [50]. Our results highlight, for the first time, a positive modulation of the BCP-CB2 axis on muscular ATP synthesis. Therefore, the reported data confirm both an insulin and physical exercise-mimetic role of this pathway on muscular glucose metabolism. AM630 and SR144528 overall inhibitory effect on the basal enzymatic activities we tested could reflect their well-known behavior as inverse agonists; this feature is dependent from the basal cannabinoid receptor activation, also detectable in the absence of agonist-induced stimulation [27].

It would be attractive, and surely the object of future investigations, to deepen the intracellular signaling pathways involved in the CB2 dependent boost of glucose metabolism in skeletal myotubes. A key element of this intracellular modulation could be the AMP kinase, the metabolic sensor that improves cellular ATP supply by enhancing biochemical pathways generating, rather than consuming, ATP [51]. As AMPK has been pointed out as a component of the CB2-dependent protective pathway in rat cortical neurons [52], its involvement in the CB2-dependent effects in skeletal muscle metabolism could also be conceivable.

## 5. Conclusions

In conclusion, this study focused on the positive role of the BCP-CB2 receptor pathway in enhancing all the steps of glucose metabolism in skeletal myotubes. In particular, the stimulatory effects were evidenced in glucose uptake, glycolytic enzymes activity, PDH, and TCA cycle enzyme activity, electron transport chain, and ATP synthesis.

Giving the established relevance of skeletal muscle glucose metabolism in the homeostatic modulation of the whole body metabolism, these results could offer a novel starting point for future supplementary approach to treat several diseases, from metabolic syndrome to skeletal muscle mass decline both due to aging and other pathological states.

## Figures and Tables

**Table 1:** *Trans-*β-Caryophyllene (BCP) effects on glycolytic pathway.

Treatments	PKF1	GAPDH	Enolase	PK
CTRL	0.2011 ± 0.0202	1.5244 ± 0.0675	3.4756 ± 0.1327	0.5411 ± 0.0322
INS	0.6422 ± 0.0257	2.4522 ± 0.0455	7.8967 ± 0.1827	1.3767 ± 0.0455
BCP	0.4378 ± 0.0201	2.1156 ± 0.0566	6.5489 ± 0.1505	1.0578 ± 0.0499
BCP + AM630	0.2511 ± 0.0184	0.5833 ± 0.0489	2.1411 ± 0.1623	0.3556 ± 0.0214
AM630	0.2278 ± 0.0138	0.6033 ± 0.0402	2.0811 ± 0.0946	0.3411 ± 0.0274
BCP + SR144528	0.2475 ± 0.0140	0.4188 ± 0.0311	2.1413 ± 0.1923	0.3113 ± 0.0246
SR144528	0.1750 ± 0.0157	0.4238 ± 0.0317	1.8925 ± 0.1796	0.2550 ± 0.0251

Values are mean ± SEM of three independent experiments. Values are expressed as nmoles NAD+/min/mg cell proteins (PFK-1, enolase, PK) or nmoles NADH/min/mg cell proteins (GAPDH).

**Table 2:** BCP effects on mitochondrial pathway.

Treatments	PDH	Citrate Synth	Aconitase	IsocitrateDH	aKGDH	SuccinateDH
CTRL	1.2111 ± 0.0723	1.0100 ± 0.0350	0.3211 ± 0.0232	1.220 ± 0.0554	0.7300 ± 0.0281	1.044 ± 0.0423
INS	1.7566 ± 0.0427	1.6955 ± 0.0490	0.5600 ± 0.0348	2.1711 ± 0.0542	1.2078 ± 0.0282	1.4644 ± 0.1284
BCP	1.3122 ± 0.0457	1.5177 ± 0.0604	0.4422 ± 0.0379	1.7855 ± 0.0513	1.0422 ± 0.0196	1.4277 ± 0.0522
BCP + AM630	0.9166 ± 0.0824	0.6133 ± 0.0455	0.2200 ± 0.0224	0.8411 ± 0.0296	0.4922 ± 0.0196	0.6944 ± 0.0278
AM630	0.9511 ± 0.0771	0.6277 ± 0.0488	0.1989 ± 0.0200	0.8266 ± 0.0493	0.4911 ± 0.0260	0.7011 ± 0.0293
BCP + SR144528	0.9837 ± 0.0997	0.5125 ± 0.0549	0.2350 ± 0.0220	0.7162 ± 0.0213	0.3738 ± 0.0260	0.6525 ± 0.0184
SR144528	0.8025 ± 0.0970	0.5150 ± 0.0534	0.2100 ± 0.0266	0.7250 ± 0.0367	0.4000 ± 0.0354	0.6612 ± 0.0384

Values are mean ± SEM of three independent experiments. Values are expressed as nmoles NADH/mg mitochondrial proteins (PDH, isocitrate dehydrogenase, αKG dehydrogenase), nmoles citrate or isocitrate/mg mitochondrial proteins (citrate synthase, aconitase), nmoles FADH2/mg mitochondrial proteins (succinate dehydrogenase).

**Table 3:** BCP effects on mitochondrial metabolism.

Treatments	ETC	ATP
CTRL	0.5722 ± 0.0305	12.5833 ± 0.6169
INS	1.0856 ± 0.0312	25.0311 ± 0.9975
BCP	0.9778 ± 0.0310	20.0367 ± 0.6576
BCP + AM630	0.3767 ± 0.0291	7.4978 ± 0.2913
AM630	0.3689 ± 0.0126	7.2322 ± 0.4469
BCP + SR144528	0.2963 ± 0.0210	7.1350 ± 0.3379
SR144528	0.3063 ± 0.0240	6.4625 ± 0.2724

Values are mean ± SEM of three independent experiments. Values are expressed as nanomoles of cytochrome c reduced/min/mg mitochondrial protein (ETC) and nmol ATP/mg mitochondrial proteins (ATP).

## Data Availability

The data presented in this study are available on request from the corresponding author.
